# Editorial: Ulcerative colitis disease therapy: Unveiling the molecular mechanisms of well-defined composition from natural plants

**DOI:** 10.3389/fphar.2023.1172160

**Published:** 2023-03-15

**Authors:** Yi Tao, Kunming Qin, Huan Li, Lihong Hu

**Affiliations:** ^1^ College of Pharmaceutical Science, Zhejiang University of Technology, Hangzhou, China; ^2^ School of Pharmacy, Jiangsu Ocean University, Lianyungang, China; ^3^ Centre of Innovation for Complementary Health Product, School of Applied Science, Temasek, Polytechnic, Singapore; ^4^ Jiangsu Key Laboratory for Functional Substance of Chinese Medicine, School of Pharmacy, Nanjing University of Chinese Medicine, Nanjing, China

**Keywords:** ulcerative colitis, natural plants, molecular mechanisms, drug delivery systems, therapy

Over the course of the past year, we have completed a Research Topic that makes it possible for researchers to focus on cutting edge science in the area of ulcerative colitis disease therapy. This Research Topic should resonate with researchers around the globe who are making efforts to keep up with the rapid pace of developments in the research domain of well-defined composition from natural plants for the treatment of ulcerative colitis disease.

Ulcerative colitis (UC) is a type of inflammatory bowel disease (IBD) that affects the mucosal and submucosal layers of the colon and rectum. Estimates of the prevalence of UC range from 7 to 246 per 100,000 individuals. The main objectives of UC therapy are, first, to induce the reduction of symptoms during acute inflammation and, second, to control chronic inflammation, thereby preventing the spread and perpetuation of the inflammatory process.

One of the main strategies to effectively neutralize the exacerbated immune response is to interfere in various stages of the inflammatory cascade, mainly with the use of corticosteroids, aminosalicylates, and immunosuppressants. In recent years, biologic therapies, including tumor necrosis factor (TNF) antagonists and the anti-α4β7 antibody vedolizumab, have improved the management of UC compared to conventional therapeutics. Unfortunately, these treatments are associated with potentially serious side effects, such as gastrointestinal problems (diarrhea and abdominal pain), anemia, hepatotoxicity, nephrotoxicity, and hypersensitivity reactions, thus limiting their chronic use. In addition, when used for a prolonged period, these therapies may represent a high cost for patients. Considering this, the development of new drug treatments that combine efficacy and safety is an important goal in UC therapy.

Researchers in the field have been working hard in search of new therapeutic strategies for UC through the application of natural products. Some examples include medicinal plants, bioactive/nutraceutical compounds of natural origin, classic prescriptions, and essential oils. Some of the studies that examine animal extract show that a thermosensitive hydrogel strategy may be valid for the prevention and treatment of UC.

UC is a high-grade inflammatory response mediated by the immune system that may be a consequence of microbial dysbiosis and disruption. The polarization imbalance of intestinal M1/M2 macrophages will lead to the dysregulation of intestinal inflammation. Yang et al. summarized the ingredients of traditional Chinese medicine, which may alleviate UC by regulating macrophage polarization. Multiple pathways, including the NF-κB signaling pathway, PPAR-γ signaling pathway, PI3K/Akt signaling pathway, MAPK signaling pathway, JAK/STAT signaling pathway, Wnt/β-catenin signaling pathway, and AMPK signaling pathway, are involved in the regulation of M1/M2 macrophages polarization.

Chang-Yan-Ning (CYN) formula (Yu et al.) and Huazhuojiedu decoction (HZJDD) (Jia et al.) are two classic traditional Chinese medicine prescriptions. Two groups adopted the network pharmacology strategy to predict their potential targets and then validated the potential mechanism of these classical prescriptions on UC. The CYN formula inhibited the protein abundance of IL17 and HIF-1α and increased PPARγ and CCL2 in the colon, as well as facilitating the alternative activation of peritoneal macrophages. Notably, the CYN formula suppressed abundance of the genus *Escherichia Shigella*, which induces diarrhea and provokes acute colitis, and increased abundance of Lachnospiraceae *NK4A136* and *Faecalibaculum rodentium*, which enhance the gut barrier function. HZJDD decoction reduced the disease activity index score, improved colon length, and effectively repaired the histomorphological and micromorphological changes of the colon. Mechanistically, the HZJDD decoction suppressed the expression of the NLRP3/caspase-1 signaling pathway at the gene and protein levels to inhibit the pyroptosis of colon cells. The above two works suggest network pharmacology is powerful for clarifying therapeutic mechanism of classic traditional Chinese medicine prescriptions.

The dextran sodium sulfate-induced murine colitis model is a routine model for UC investigation. In the work of He et al., the fruit fly *Drosophila melanogaster* was adopted as a model to investigate the function and regulatory mechanism of *Astragalus membranaceus* extract on intestine disruption induced by SDS. Compared to rodent models, the merits of the fruit fly model include less ethical concerns, low maintenance costs, and short generation time. The work identified seven bioactive compounds, including formononetin, isoliquiritigenin, isorhamnetin, astragaloside I, astragaloside III, vanillic acid, and caffeic acid, which can prevent SDS-induced inflammatory injury. This work also proved that fruit fly is an ideal and economical model for the rapid screening of therapeutically natural products for UC.

Chemotherapeutic agents such as 5-fluorouracil (5-FU) can cause intestinal mucosal injury. Zheng et al. discovered that the essential oil of *Brucea javanica* (BJO) could ameliorate 5-FU-induced epithelial apoptosis through the downregulation of Bax and caspase-3 and the upregulation of Bcl-2. The protective effects of BJO in chemotherapeutic intestinal mucosal injury are most likely attributable to the activation of Nrf2/HO-1. This work suggested that BJO may serve as a promising therapeutic choice for 5-FU-induced ulcerative colitis.

The aforementioned studies have mainly focused on the mechanism of therapeutic agents for UC. Novel drug delivery systems have also been increasingly explored for treating UC. Wu et al. synthesized thermosensitive enema gels by loading them with extracts of *Periplaneta Americana* dried insects, which can transform from a viscous liquid state to gel *in situ* upon exposure to changing temperatures. The thermosensitive enema gels showed obvious effects on inhibiting the release of inflammatory cytokines both *in vivo* and *in vitro*. The protective effect of extracts of *Periplaneta Americana* was found to be associated with the regulation of necroptosis mediated by the RIP1/RIP3/MLKL signaling pathway. The rectal route has several advantages, including low susceptibility to enzymatic degradation and the ability to bypass the hepatic first-pass effect. This work proved that thermosensitive enema gels are efficient drug delivery systems for treating UC.

